# Effect of CGRP-Adenoviral Vector Transduction on the Osteoblastic Differentiation of Rat Adipose-Derived Stem Cells

**DOI:** 10.1371/journal.pone.0072738

**Published:** 2013-08-30

**Authors:** Zhong Fang, Qin Yang, Wei Xiong, Guang-hui Li, Hui Liao, Jun Xiao, Feng Li

**Affiliations:** 1 Department of Orthopedics, Tongji Hospital, Tongji Medical College, Huazhong University of Science and Technology, Wuhan, Hubei, P R China; 2 Department of Pathology, Tongji Hospital, Tongji Medical College, Huazhong University of Science and Technology, Wuhan, Hubei, P R China; National Institutes of Health, United States of America

## Abstract

Calcitonin gene-related peptide (CGRP) promotes osteoblast recruitment and osteogenic activity. However, no evidence suggests that CGRP could affect the differentiation of stem cells toward osteoblasts. In this study, we genetically modified adipose-derived stem cells (ADSCs) by introducing the CGRP gene through adenoviral vector transduction and investigated on cellular proliferation and osteoblast differentiation *in vitro* and osteogenesis *in vivo* as well. For the *in vitro* analyses, rat ADSCs were transducted with adenoviral vectors containing the CGRP gene (Ad-CGRP) and were cultured in complete osteoblastic medium. The morphology, proliferative capacity, and formation of localized regions of mineralization in the cells were evaluated. The expression of alkaline phosphatase (ALP) and special markers of osteoblasts, such as Collagen I, Osteocalcin (BPG) and Osteopontin (OPN), were measured by cytochemistry, MMT, RT-PCR, and Western blot. For the *in vivo* analyses, the Ad-CGRP-ADSCs/Beta-tricalcium phosphate (*β*-TCP) constructs were implanted in rat radial bone defects for 12 weeks. Radiography and histomorphology evaluations were carried out on 4 weeks and 12 weeks. Our analyses indicated that heterogeneous spindle-shaped cells and localized regions of mineralization were formed in the CGRP-transduced ADSCs (the transduced group). A higher level of cellular proliferation, a high expression level of ALP on days 7 and 14 (p<0.05), and increased expression levels of Collagen I, BPG and OPN presented in transduced group (p<0.05). The efficiency of new bone formation was dramatically enhanced *in vivo* in Ad-CGRP-ADSCs/*β*-TCP group but not in β-TCP group and ADSCs/*β*-TCP group. Our results reveal that ADSCs transduced with an Ad-CGRP vector have stronger potential to differentiate into osteoblasts *in vitro* and are able to regenerate a promising new tissue engineering bone *in vivo.* Our findings suggest that CGRP-transduced ADSCs may serve as seed cells for bone tissue engineering and provide a potential way for treating bone defects.

## Introduction

Bone is a highly vascularized and innervated connective tissue that is subject to continuous remodeling and renovation. Many complex clinical conditions require bone regeneration in large quantities, such as large bone defects, atrophic nonunions and osteoporosis [1]. Bone tissue engineering is a promising treatment for these diseases [2]. Furthermore, genetically-modified, engineered bone tissue is an attractive approach with great potential for repairing large bone defects or nonunions [3]–[5].

Mesenchymal stem cells (MSC) hold great promise for future translational research and clinical applications in many fields. Much research has focused on bone mesenchymal stem cell (BMSCs) isolated from bone marrow; however, harvesting bone marrow causes considerable discomfort to the patient and yields a relatively small number of cells. In contrast, adipose tissue provides an abundant and easily accessible source of adult stem cells, namely adipose-derived stem cells (ADSCs). ADSCs have the ability to differentiate equally along multiple lineage pathways into fat, bone, cartilage, skeletal muscle, smooth muscle, cardiac muscle, endothelial cells, hematopoietic cells, hepatocytes, and neuronal cells [6]–[8]. Animal and clinical studies have shown that ADSCs are capable of repairing damaged skeletal tissue or large-bone segmental defects [9], [10].

Another critical factor for bone tissue engineering is growth factors. Many previous studies have focused on bone morphogenetic proteins (BMPs) [11], [12]. However, recent research observed that calcitonin gene-related peptide (CGRP) contains CGRP immunoreactive nerve fibers directly involved in the local regulation of bone remodeling [13]–[15]. Further evidence showed that CGRP plays a role in bone metabolism. Transgenic mice over-expressing CGRP in osteoblasts increased bone density by increasing bone formation [16] whereas mice lacking CGRP expression displayed osteopenia, caused by decreased bone formation [17]. Although numerous investigators have identified CGRP’s stimulatory effects in osteoblasts, no previous studies have examined the osteogenic effects of CGRP in MSCs.

Our goal was to determine whether genetic modification of rat ADSCs to cause CGRP over-expression would enliven the stem cells, enable them to differentiate into osteoblasts and enhance osteogenic capacity *in vitro* and *in vivo*. From these results, we further speculate that CGRP-modified ADSCs may be effective seed cells in tissue engineering to promote the healing of bone defects.

## Materials and Methods

Fetal bovine serum (FBS), trypsin, Dulbecco’s modified Eagle’s medium (DMEM) and Lipofectamine 2000 were purchased from Invitrogen, USA. PCR primers, Taq DNA polymerase, DNA ladder and oigo dT were obtained from Sangon, China. The *Pme*I, *Pac*I, and *HindIII* restriction enzymes were provided by NEB. The plasmid DNA extraction (Mini) Kit was provided by QIAGEN, UK. Escherichia coli strain DH5a and the AdEasy Vector System were provided by GeneChem, China. HEK293T cells (ATCC#: CRL-11268) were used to generate adenoviral particles. Beta-tricalcium phosphate (*β*-TCP) scaffolds were purchased from Beta Whitlockite Plasma Biotal (Tideswell, UK). Sprague-Dawley rats were from the Experimental Animal Center of Tongji Medical College and were used following protocols approved by the Animal Care and Use Committee of Tongji Medical College of Huazhong University of Science and Technology (Permit Number: 20051007).

### Construction of Plasmid Vectors and Adenoviral Particles

The AdEasy Vector System was used to construct the pAd-EGFP adenoviral vector. This vector contained the EGFP reporter gene derived from pEGFP-C. The transfer vector pShuttle-CGRP was constructed using standard methods. pShuttle-CGRP was linearized with *Pme*I and co-transformed into the competent *E. coli* strain DH5a along with pAdeasy-1, the viral DNA plasmid. Briefly, 1 µg of linearized recombinant transfer vector pShuttle- CGRP (5 µL) and 1.0 µL of pAdEasy-1 vector (100 µg/µL) were added to 200 µL of competent- DH5a cells in a 14-mL culture tube. These components were mixed gently, incubated on ice for 1 h, heat-shocked at 42°C for 1 min and then immediately returned to ice for 5 min. Subsequently, 1000 µL of LB media were added, and the cells were shaken (280 r/min) and incubated for 1 h at 37°C. Cells were plated onto 100 mm Petri dishes with LB agar and incubated overnight at 37°C. The recombinant clones (pAd5-CGRP) were identified by restriction enzyme analysis.

pAdEasy-1 lacks E1 and E3, and its E1 function can be complemented in HEK293 cells. The recombinant adenoviral construct, pAd5-CGRP, was then cleaved with *Pac*I to expose inverted terminal repeats and transfected HEK293 cells to produce viral particles. The Ad5-CGRP construct was purified through two cesium chloride gradients, and the purified virus was desalted by dialysis at 4°C against 10 mmol/L Tris-HCl buffer with 4% sucrose. Virus was stored in aliquots in liquid nitrogen. Viral titer was determined using Adeno-X™ Rapid Titer Kits.

### Isolation, Culture and Genetic Modification of ADSCs

The isolation and cell culture of rats ADSCs were described previously [8].To achieve high rates of viral infection, we used a protocol involving two centrifugation steps. Cells from sub-confluent cultures were harvested by treatment with 0.05% (w/v) EDTA in phosphate-buffered saline (PBS) containing MgCl_2_, CaCl_2_ and 0.25% (w/v) trypsin. Cells were seeded at a density of 100,000 cells/cm^2^ and centrifuged at 1000×g at 37°C for 10 min. The concentrated virus preparation was diluted 1∶1.5 with DMEM medium and applied to the pre-centrifuged cells, which were then incubated at 37°C for 40 min before a second centrifugation for 60 min was performed. The infected cells were incubated under standard conditions overnight, followed by a medium change. EGFP reporter gene expression was analyzed at 24 h, 48 h, and 72 h after transduction. The CGRP transduced and untransduced ADSCs were classified as the transduced group and control group, respectively. All experiments and cell number determinations were performed in triplicate.

### Osteogenic Differentiation of ADSCs *in vitro*

After three passages of culture expansion, the cells were trypsinized and replated onto 6-well tissue culture plates at 10^5^ cells per well. Cells were allowed to adhere and grow for 3 days, and then the medium was replaced with osteogenic medium containing DMEM with 10% FBS, 0.1 M dexamethasone (Sigma, USA), 10 mM glycerol phosphate (Sigma, USA), and 50 g/ml ascorbic acid-2-phosphate (Sigma, USA). The medium was changed every 3 days.

### Morphology, Growth Curve and Doubling Time of ADSCs after Osteogenic Induction

Changes in cell morphology of CGRP-ADSCs and untransduced ADSCs plated at a cell density of 2×10^4^/ml were observed under an inverted microscope after osteogenic induction for 3 days and 7 days. Meanwhile, the growth curve of the two groups was plotted by MTT assay, and the doubling time was calculated using the formula: D_t_ = Δt (T_2_−T_1_) In_2_/(InN_2_−InN_1_), where Δt represent days of duration, T_1_ and T_2_ represent time, and N_1_,N_2_ equal number of cells.

### Alkaline Phosphatase (ALP) Staining and Activity Assays

Osteogenesis was assessed by ALP expression using the von Kossa staining method described previously [18] and by ALP activity assays at days 7 and 14 after the initial osteogenic induction. ALP activity was detected using ALP detection kits (Pierce Biotechnology, Rockford, IL). In brief, ADSCs were lysed with 1% Triton-X (Sigma-Aldrich, St. Louis, MO). The AP activity of each cell lysate was determined using pNPP Reagent (Moss Inc., Pasadena, MD); the reaction was incubated until significant color developed, and absorbance was read at 490 nm. The amount of 4-nitrophenol (4-NP) produced per minute was determined relative to A490 readings of a standard curve generated using serial dilutions of 4-NP (Sigma-Aldrich, St. Louis, MO). ALP activity from each well was normalized to total protein, which was quantified using a BCA Protein Assay Kit.

### Mineralization in Induced, Transduced Cells Identified by Tetracycline Notation

Osteogenesis was also assessed using a new mineralization staining technique with tetracycline notation. In brief, osteogenesis was induced in cells cultured in a 6-well plate for 7 or 14 days, and the cells were washed three times with PBS to remove the culture medium. Then tetracycline (50 µg/ml) was added to each well for 30 minutes. Next, cells were incubated in medium containing 10% FBS for 30 minutes, rinsed with PBS and immersed in 95% ethanol for 10 minutes. Calcified nodules were observed under an inverted fluorescence microscope.

### Detection of CGRP Expression by Immunocytochemistry

The expression of CGRP was detected by immunocytochemical staining. Briefly, after induction for 3 or 7 days, cells were fixed with 10% formalin and washed with PBS. The fixed cells were permeabilized with 1% NP-40 and blocked with 10% FBS, followed by an incubation with CGRP antibody (Santa Cruz Biotechnology, Santa Cruz, CA, USA) for 1 h. After washing, cells were incubated with biotin-labeled secondary antibody for 30 minutes, followed by incubating cells with streptavidin–horseradish peroxidase (HRP) conjugate for 20 minutes at room temperature. The presence of the expected protein was visualized by 3,3′-Diaminobenzidine (DAB) staining, and cells were examined using the Quantimet 500 image analysis system (Leica Co, Germany). Staining without the primary antibody or with control IgG was used as a negative control.

### RT-PCR and Western Blot Analysis to Detect Expression of the Target Gene and Osteoblast Markers

Total RNA was extracted from different samples using Trizol reagent. RT-PCR was performed to detect the expression of the target gene (CGRP) and osteoblast marker genes (Collagen I, BPG, and OPN). Glyceraldehyde-3-Phosphate Dehydrogenase (GAPDH) served as a housekeeping gene. PCR was carried out with the primers listed in Table 1. PCR cycling for CGRP used the following conditions: 1 cycle at 95°C for 1 min; 30 cycles consisting of 30 s at 95°C, 55°C for 10 s, and 72°C for 40 s; and 1 cycle at 72°C for 5 min. PCR cycling for Collagen I, BPG, and OPN used the following conditions: 1 cycle at 94°C for 1 min; 30 cycles at 94°C for 30 s, 55°C for 10 s, and 72°C for 40 s; and 1 cycle at 72°C for 5 min. Finally, the PCR product was visualized on a Bio-Imaging System (Bio-Rad Co, USA) and the ratio of target gene to GAPDH was calculated to indicate relative levels.

**Table 1:** Primers used for real-time PCR analysis.

Gene	Primers	Annealing temperature (°C)
CGRP	Forward: 5′-ACGATGCCGCCATTTGTG-3′ Reverse: 5′-CGCCTCGCCTTCTTCAGT-3′	62
Collagen I	Forward: 5′-GAGCGGAGAGTACTGGATCG-3′ Reverse: 5′-GCTTCTTTTCCTTGGGGTTC-3′	58
OPN	Forward: 5′-GACGGCCGAGGTGATAGCTT-3′ Reverse: 5′-CATGGCTGGTCTTCCCGTTGC-3′	63
BPG	Forward: 5′-AAAGCCCAGCGACTCTC-3′ Reverse: 5′-CTAAACGGTGGTGCCATAGAT-3′	59
GAPDH	Forward: 5′-AACCCATCACCATCTTCCAGG-3′ Reverse: 5′-GCCTTCTCCATGGTGGTGAA-3′	60

The cells were washed twice with ice-cold phosphate-buffered saline (PBS) and directly lysed in Laemmli buffer. The lysate was sonicated, boiled for 5 minutes and centrifuged at 16,000 g for 10 minutes at 4°C. The supernatant was recovered as total cell lysate, aliquoted and stored at −80°C. Equal amounts of protein (10 µg) were separated on 8% SDS-PAGE and electro-transferred to 0.45 µm polyvinylidene difluoride membranes (Millipore, Bedford, USA). Following transfer, membranes were blocked with a solution of 0.1% Tween 20/TBS (TBS/T) containing 5% non-fat milk for one hour at room temperature and then incubated overnight at 4°C with monoclonal mouse anti-human CGRP and OPN antibodies (GeneTex, USA, final dilution 1∶300) or rabbit polyclonal anti-human Collagen I and BPG antibodies (Chemicon, Temecula, California, USA, final dilution 1∶400). The bands were visualized by nitroblue tetrazolium/5-bromo-4- chloro-3-indolyl-phosphate. GAPDH served as an endogenous control. For densitometric analyses, blots were scanned and quantified using Quantity One analysis software (Bio- Rad, Hercules, CA, USA). The results were expressed as the percentage of GAPDH immunoreactivity.

### Osteogenic Differentiation of ADSCs *In vivo*

#### Cell Seeding on scaffolds

Ad-CGRP-ADSCs and ADSCs as control were collected and seeded at a density of 1×10^6^ in 30 µL on the *β*-TCP scaffolds and incubated at 37°C for 2 h to allow cells to attach to the scaffolds, respectively. Then the scaffolds were transferred into a 96-well plate. Each well was cultured with the osteogenic medium for 7 days. The medium was changed every 2–3 days after cell seeding on *β*-TCP.

### Preparation of Bone Defect Model and Implantation

Thirty healthy Sprague-Dawley rats weighting about 250g each were divided into three groups for the implantation of *β*-TCP (Group 1, n = 10), ADSCs/*β*-TCP (Group 2, n = 10), and Ad-CGRP-ADSCs/*β*-TCP (Group 3, n = 10). Rats were anesthetized with 2% isoflurane inhalation for the duration of the procedure, and a 3-mm segmental osteoperiosteal defect was created in the radius with a mini-oscillating saw bilaterally. After resection, the scaffold or cell-scaffold constructs were placed into the defects via a tight vertical pressure.

### Radiography and Histological Detection

The rats were examined by X-ray sequentially after operation of 2, 4, and 12 weeks. To quantify the analysis and compare the differences in new bone formation, bone union and remodeling of the bone defect between the two groups, we used a modified Lane and Sandhu X-ray scoring systems [19].

At four and twelve weeks postoperatively, five rats from each group were harvested and the radius with ulnas were explanted. After being fixed, decalcified, dehydrated, and embedded in paraffin wax, the tissues were cut into 7-µm sections and stained with haematoxylin and eosin (HE). For morphometric analysis, five sequential sections per implant were selected for evaluation under low magnification, allowing coverage of the entire implant. Using a Leica-Qwin 3.2 Image Analysis System (Leitz DMRD, Leica Microsystems, Inc., Bannockburn, IL, USA), all slides were evaluated by two independent observers to identify the type of bone tissue or scaffolds. The extent of bone formation or residual scaffold was indicated by the percentage of the bone tissue area or scaffold area within the defect site and an average value was calculated for each implant.

### Statistical Analysis

Each experiment was repeated three times. All data are represented as the mean ± SD, and statistical analysis was carried out using the SPSS software package (Version 12.0). Data were analyzed using the independent-samples t-test and a paired t-test. CGRP data were not normally distributed and were therefore tested using the Wilcoxon signed-rank test. p<0.05 was considered statistically significant.

## Results

### Characterization of ADSC Morphology and Growth after Transduction with Ad-CGRP

**Figure 1:**
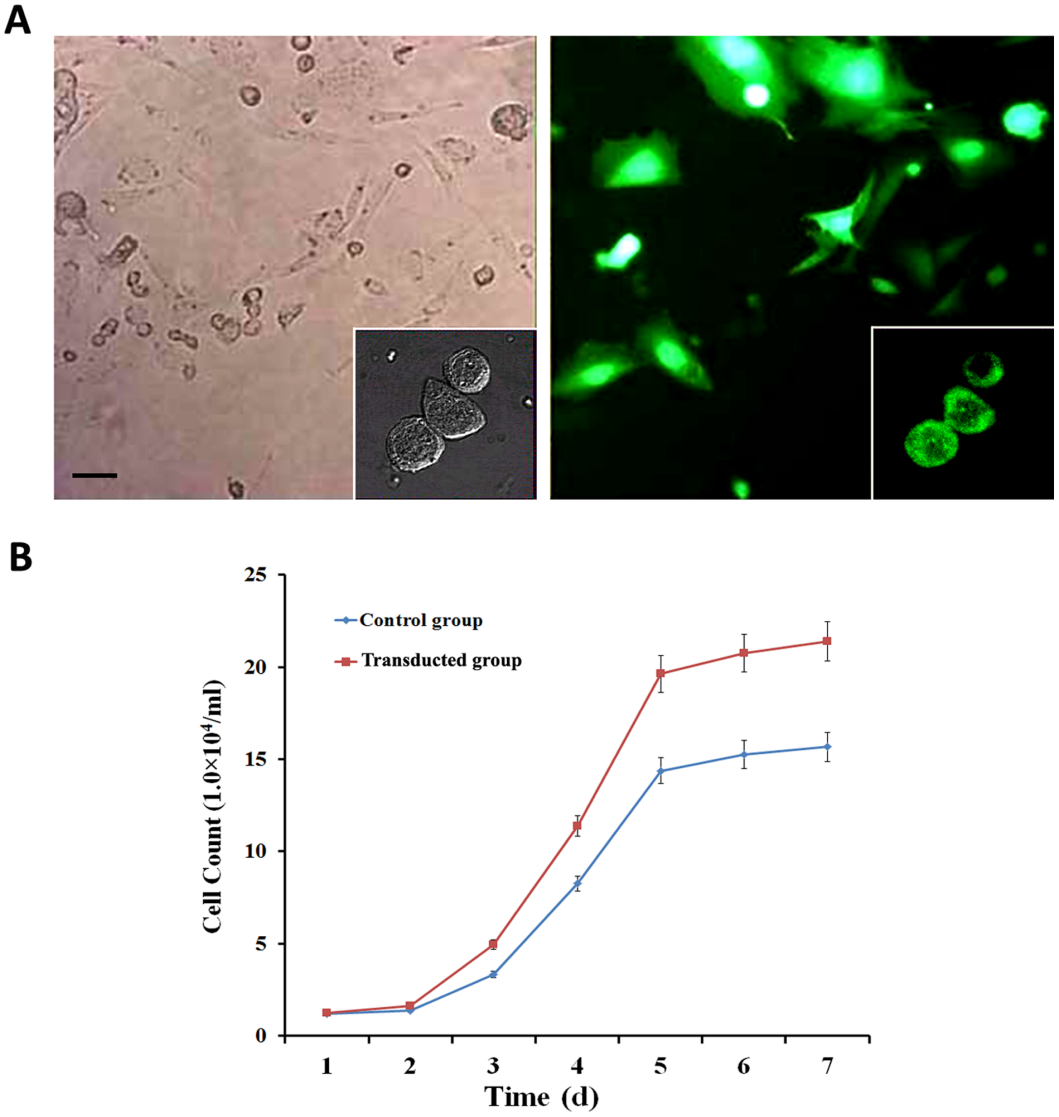
Transduction efficiency and growth curves of ADSCs. **A)** CGRP-transduced ADSCs exhibited bright green EGFP fluorescence under fluorescent microscope and indicated a transduction efficiency of approximately 81%. Scale bar equals 100 µm. **B)** The growth curves of different groups showed that the transduced group had a significantly shorter doubling time than the untransduced group. *p<0.05, transduced group vs. control group.

The CGRP-transduced ADSCs exhibited bright green EGFP fluorescence under the fluorescent microscope (**Fig. 1A**). Using adenoviral mediated-transfection, the transfection efficiency was nearly 81%. The CGRP-ADSCs appeared as heterogeneous population of spindle-shaped cells; despite some colony growth mode, while the growth was evenly distributed, long spindle-shaped cells dominated. However, the untransduced ADSCs grew only as a monolayer of flat fibroblast-like cells. Meanwhile, the proliferative capacity of each group was calculated and displayed on growth curves (**Fig. 1B**). The doubling time of transduced ADSCs was significantly shorter than the control group (*p*<0.05).

### Expression of CGRP in the ADSCs after Transduction with Ad-CGRP

**Figure 2:**
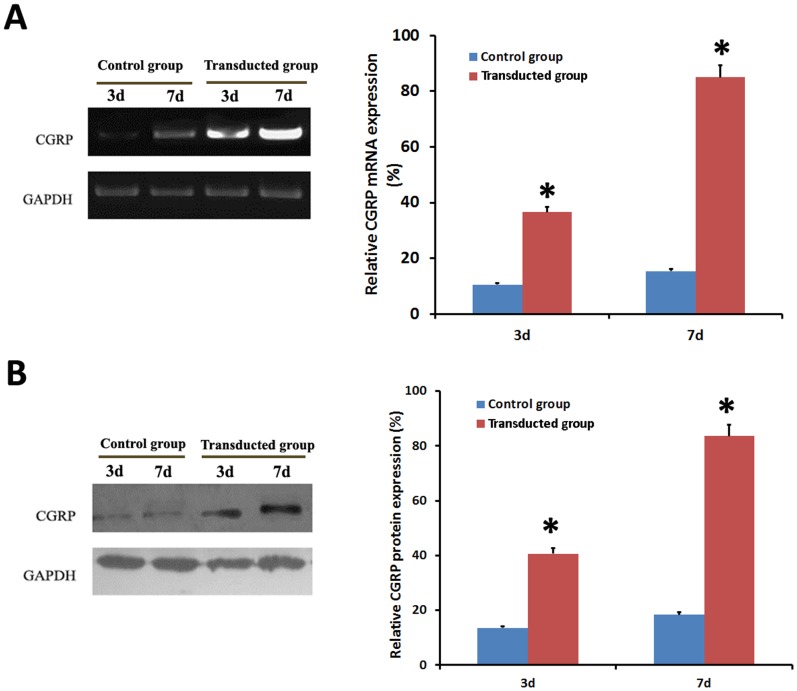
Expression of CGRP among all groups was detected by RT-PCR and Western blot. **A), B)** demonstrated higher expression of CGRP in the transduced group than the control group at 3 d or 7 d after transduction by RT-PCR and Western blot detections. *p<0.05, transduced group vs. control group.

**Figure 3:**
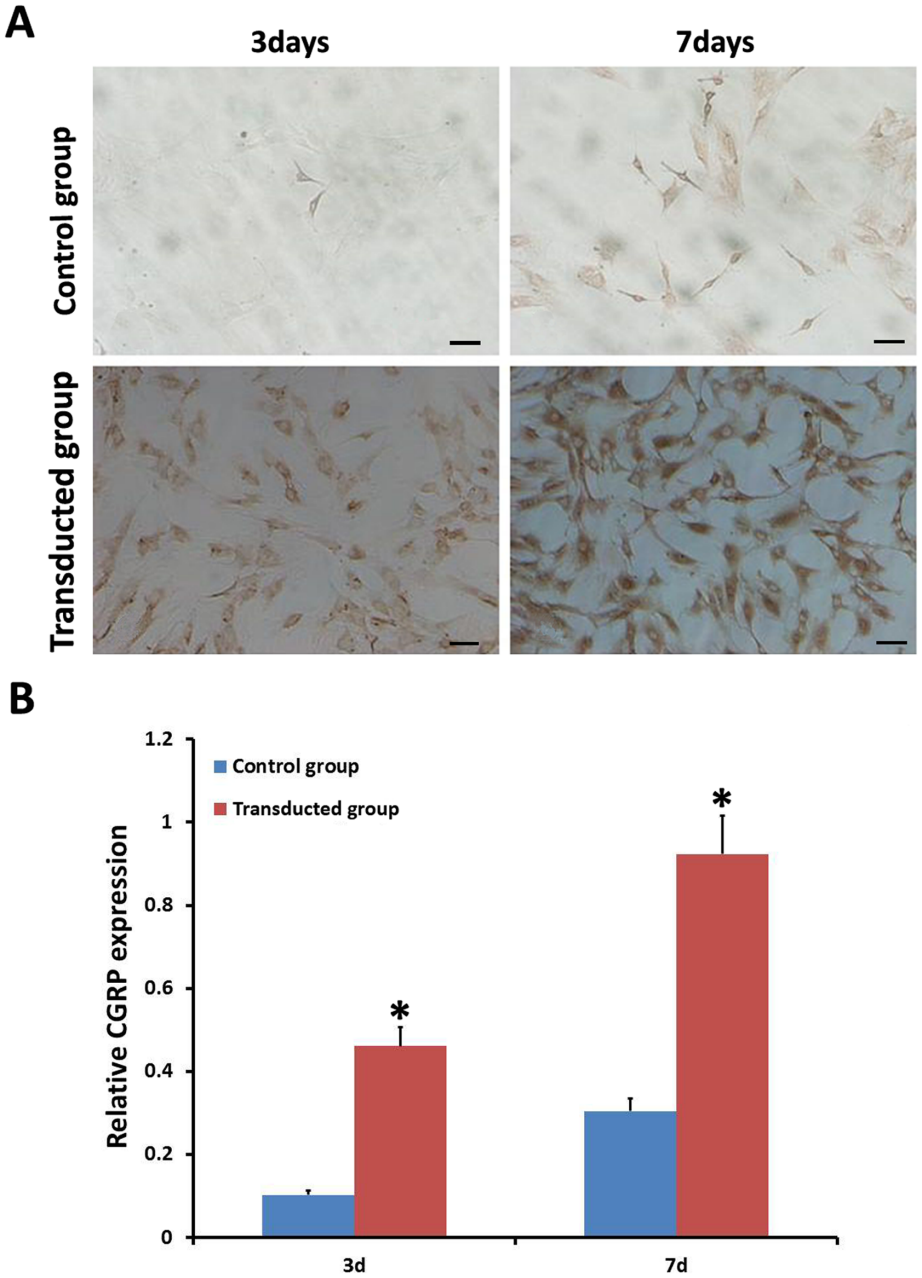
Expression of CGRP among all groups was detected by immunocytochemistry. **A), B)** Expression of the CGRP gene in the transduced group showed higher secretion of CGRP protein than in the control group after 3days and 7days. Scale bar equals 100 µm. *p<0.05, transduced group vs. control group.

To fully characterize the expression of CGRP between the two groups, RT-PCR, Western blot and immunocytochemistry on days 3 and 7 were performed. As shown in **Fig. 2A** and **Fig. 2B**, the transduced group exhibited significantly higher expression of CGRP than the control group (P<0.05) by both RT-PCR and Western blot. Similarly, immunocytocheical analyses also showed that the transduced group secreted significantly more CGRP protein than the control group by the days 3 and 7 (**Fig. 3**).

### Expression of ALP and Mineralized Nodule Formation after Osteoblast Induction

**Figure 4:**
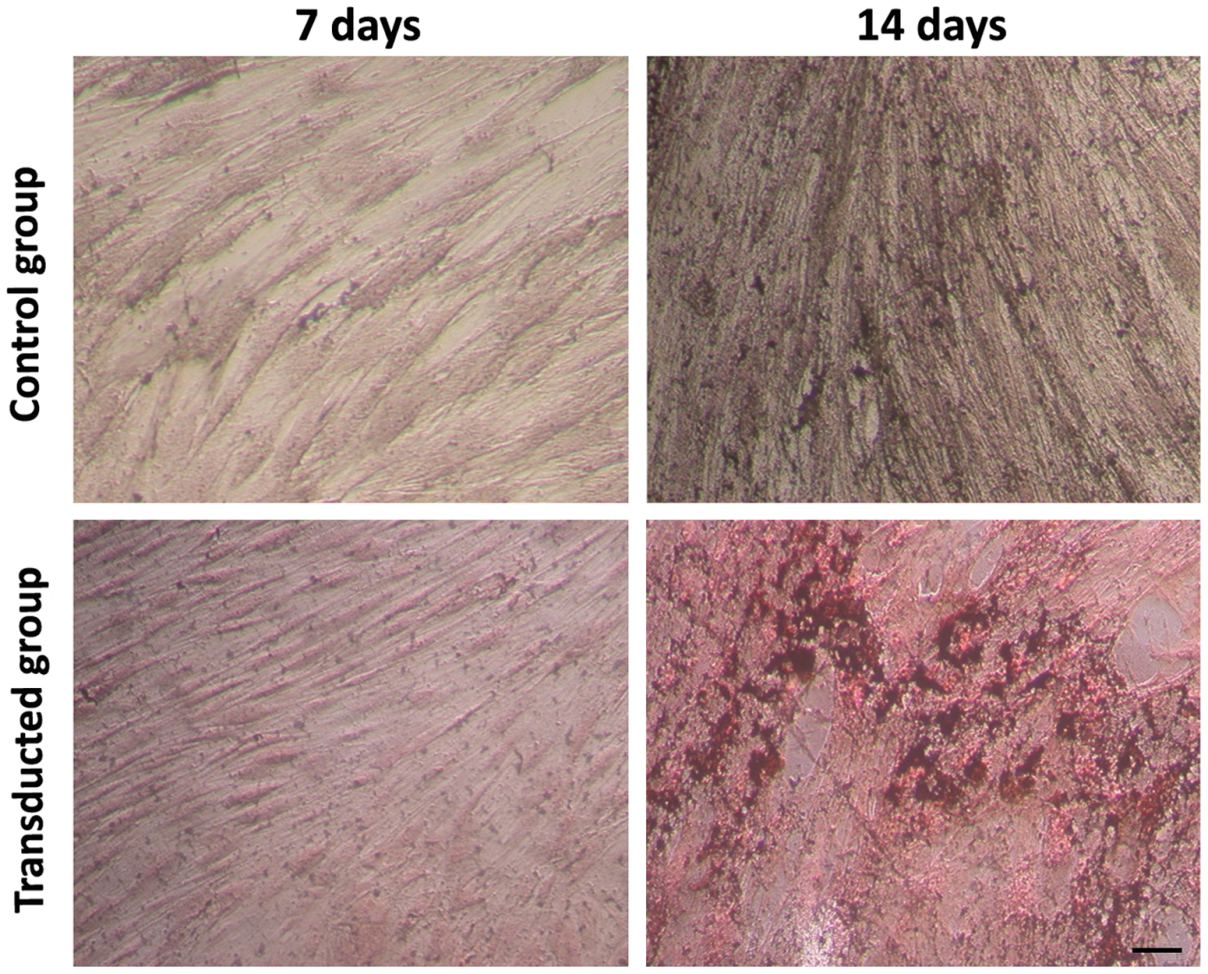
Expression of ALP after osteoblast induction. Detection of ALP staining in the transduced group displayed more red ALP-positive granules in the cytoplasm than the control group on days 7 and 14. Scale bar equals 100 µm.

To fully characterize the differentiated ADSCs after osteoblast induction, ALP activity was detected on days 3, 7, 10 and 14 (**Table 2**). Although ALP activity in both groups on days 3 was similar, ALP activity was higher in the transduced group (*p*<0.05), at approximately 1.8-fold, 1.99-fold, and 2.1-fold on days 7, 10, and 14, respectively, compared to the control group. Similarly, as shown in **Fig. 4**, ALP staining in the transduced group displayed more red ALP-positive granules in the cytoplasm than the control group on either days 7 or 14.

**Table 2:** Levels of ALP (U/L, ± s) in transduced and control cells.

Groups	3 d	7 d	10 d	14 d
Transduced group	0.94±0.31	1.95±0.24	2.88±0.40	4.65±0.23
Control group	0.91±0.42	1.28±0.41*	1.54±0.38*	2.32±0.34*

* *p<0.05,* transduced group vs. control group.

**Figure 5:**
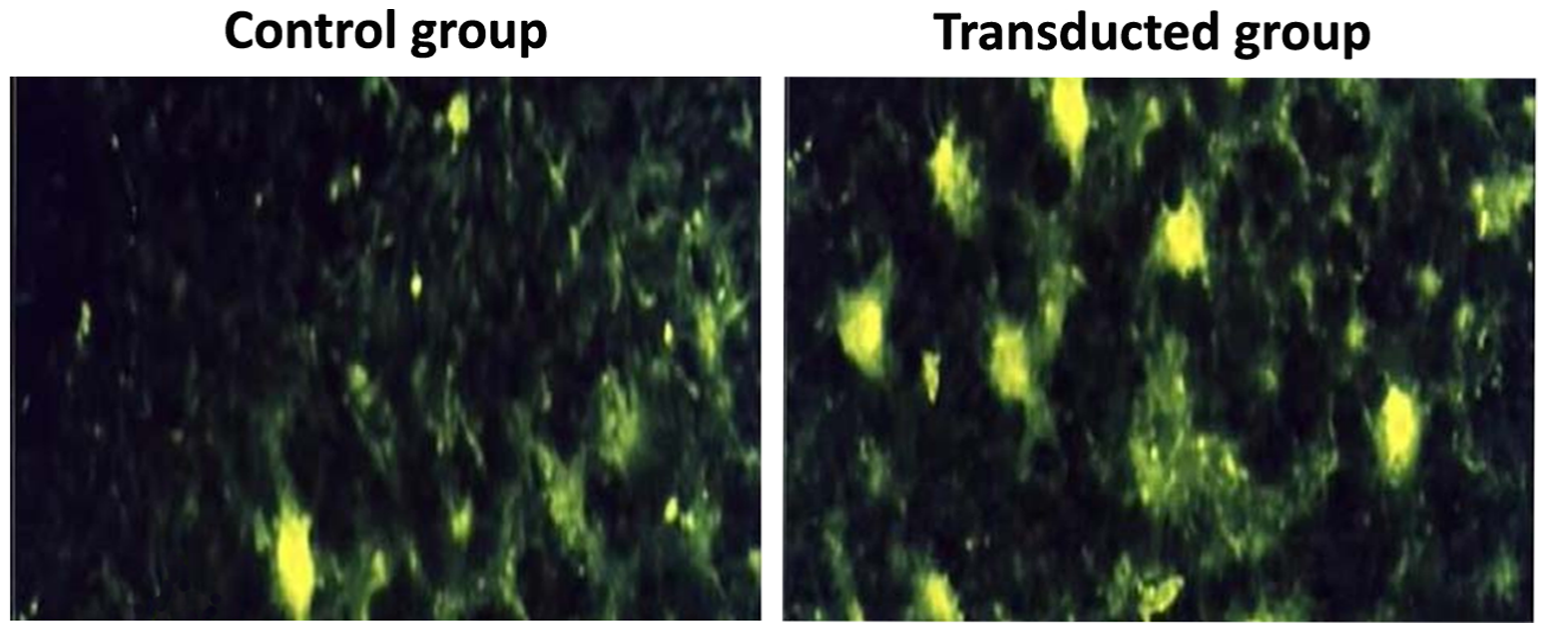
Mineralized nodule formation after osteoblast induction. Mineralized nodule formation was detected on days 7 and 14. The transduced group indicated more mineralized nodules which were display as circular, golden brown nodules formed from the combination of f tetracycline and calcium. Scale bar equals 100 µm.

To examine osteoblast differentiation, mineralized nodule formation was detected on days 7 and 14. As shown in **Fig. 5**, mineralized nodules were revealed as circular, golden brown, complex nodes, which were formatted with combination of tetracycline and calcium. The amount of mineralized nodules formed in the transduced group was significantly more than those formed in the control group (*p*<0.05) on days 14.

### Expression of Osteoblast Markers in Differentiated Ad-CGRP ADSCs

**Figure 6:**
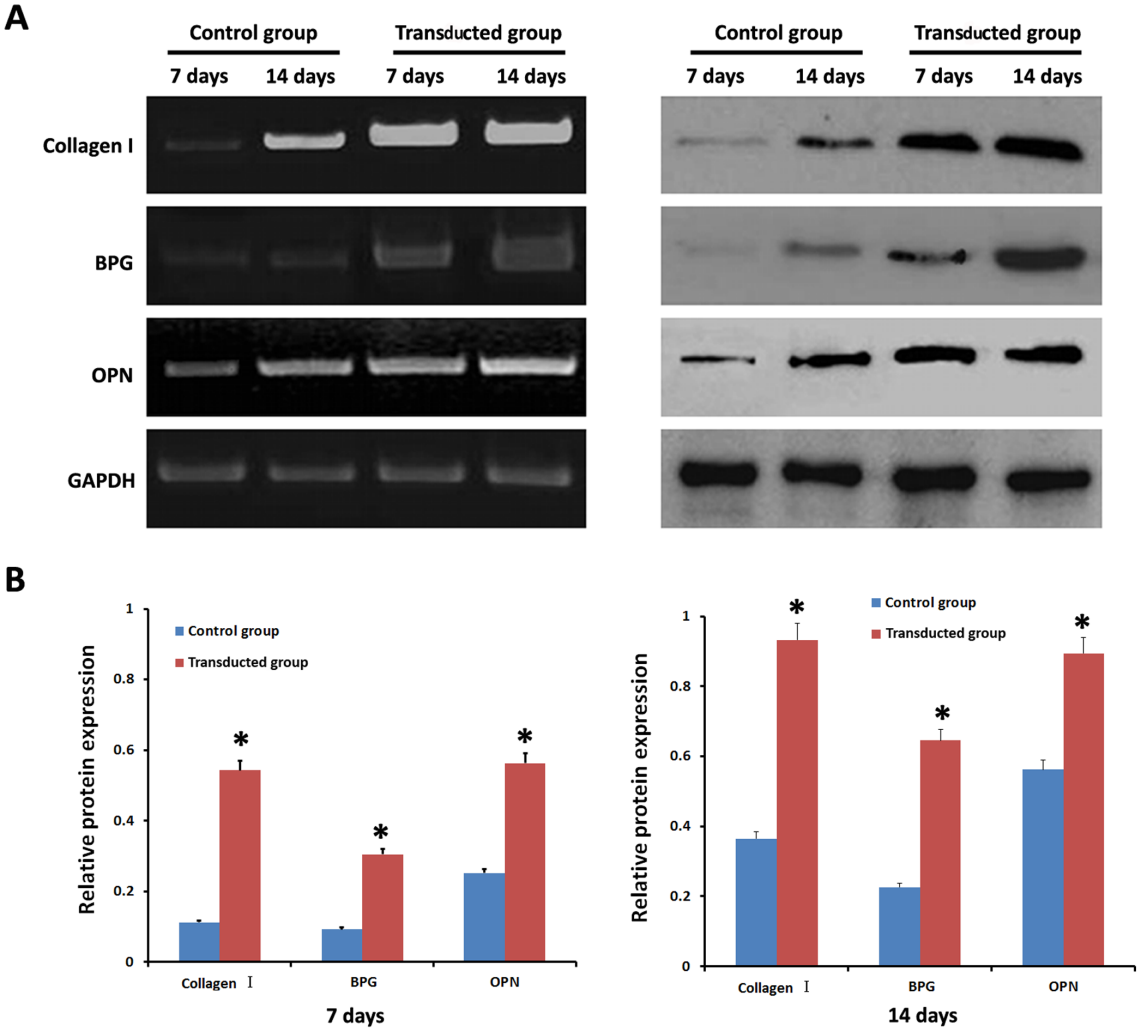
Expression of osteoblast markers in differentiated ADSCs. **A), B)** RT-PCR and Western blot showed that Collagen I, BPG and OPN were expressed at significantly higher levels in the transduced group than the control group after 7 d and 14 d. *p<0.05, transduced group vs. control group.

To fully analyze the differentiated ADSCs after osteoblast induction, RT-PCR and Western blot for markers of osteoblast lineages, such as Collagen I, BPG, and OPN, were performed on days 7 and 14. Compared to the control group, the Collagen I bands detected by RT-PCR in the transduced group were significantly brighter on days 7 and 14 (*p*<0.05) **(** **Fig. 6A** **,** **Fig. 6B** **)**. Similarly, the Collagen I bands in the transduced group using Western blot detection were significantly darker than the control group (*p*<0.05) **(** **Fig. 6A** **,** **Fig. 6B** **)**. Furthermore, the expression of BPG and OPN in the transduced group was significantly higher than the control group (*p*<0.05) by both RT-PCR and Western blot, similar to Collagen I expression **(** **Fig. 6A** **,** **Fig. 6B** **)**.

### Radiography Observation in Bone Defect Model

**Figure 7:**
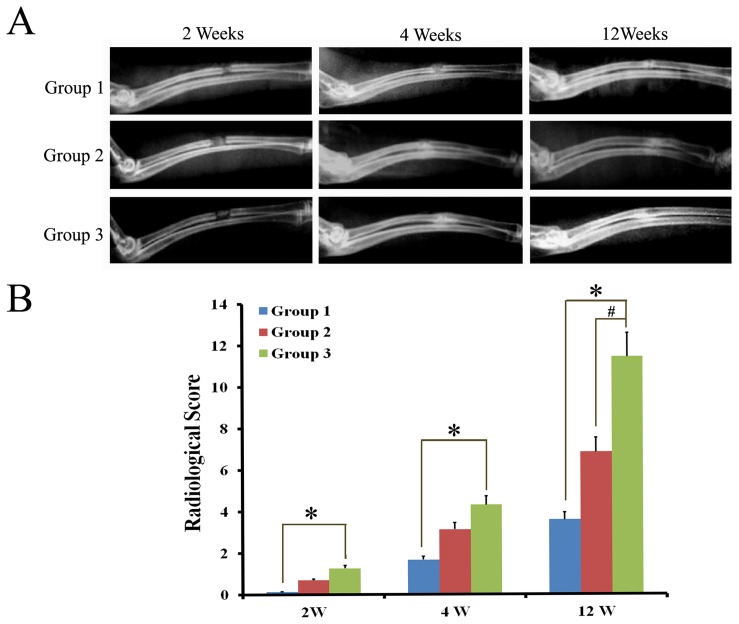
Radiography observation in bone defect model. **A)** X-rays photograph of rat radical defects in different groups at 2 weeks, 4 weeks, and 12 weeks showing complete bridging of the defect in group 3, however, a radiolucent line was still present at the defect site in group 1 at 12 weeks. **B)** Radiographical scoring also showed that new bone formation in group 3 was more significant difference than the other groups at 12 weeks (p<0.05). (Group 1 referred to *β*-TCP group; Group 2 referred to ADSCs/*β*-TC group; Group 3 referred to Ad-CGRP-ADSCs/*β*-TCP group).

As shown in **Fig 7A**, at 2 weeks, there was no markedly newly formed bone tissue in each group. At 4 weeks, there was partly newly formed high-density tissue in the defects of all groups, and the amount of the new tissue was more in the Ad-CGRP-ADSCs/*β*-TCP constructs than those treated with either the of *β*-TCP or ADSCs/*β*-TCP. At 12 weeks, there was incremental high-density tissue in *β*-TCP and ADSCs/*β*-TCP constructs. But the bone defects were not healed completely, especially in *β*-TCP group. However, in Ad-CGRP-ADSCs/*β*-TCP group, the radial defect has been completely healed. Furthermore, radiographical scoring also suggested that the bone formation in group 3 was statistically superior to that in group 1 either at 2 weeks, 4 weeks or 12 weeks (*p*<0.05; **Fig. 7B**), and also significant difference than group 2 at 12weeks (*p*<0.05; **Fig. 7B**).

### Histological Observation in Bone Defect Model

**Figure 8:**
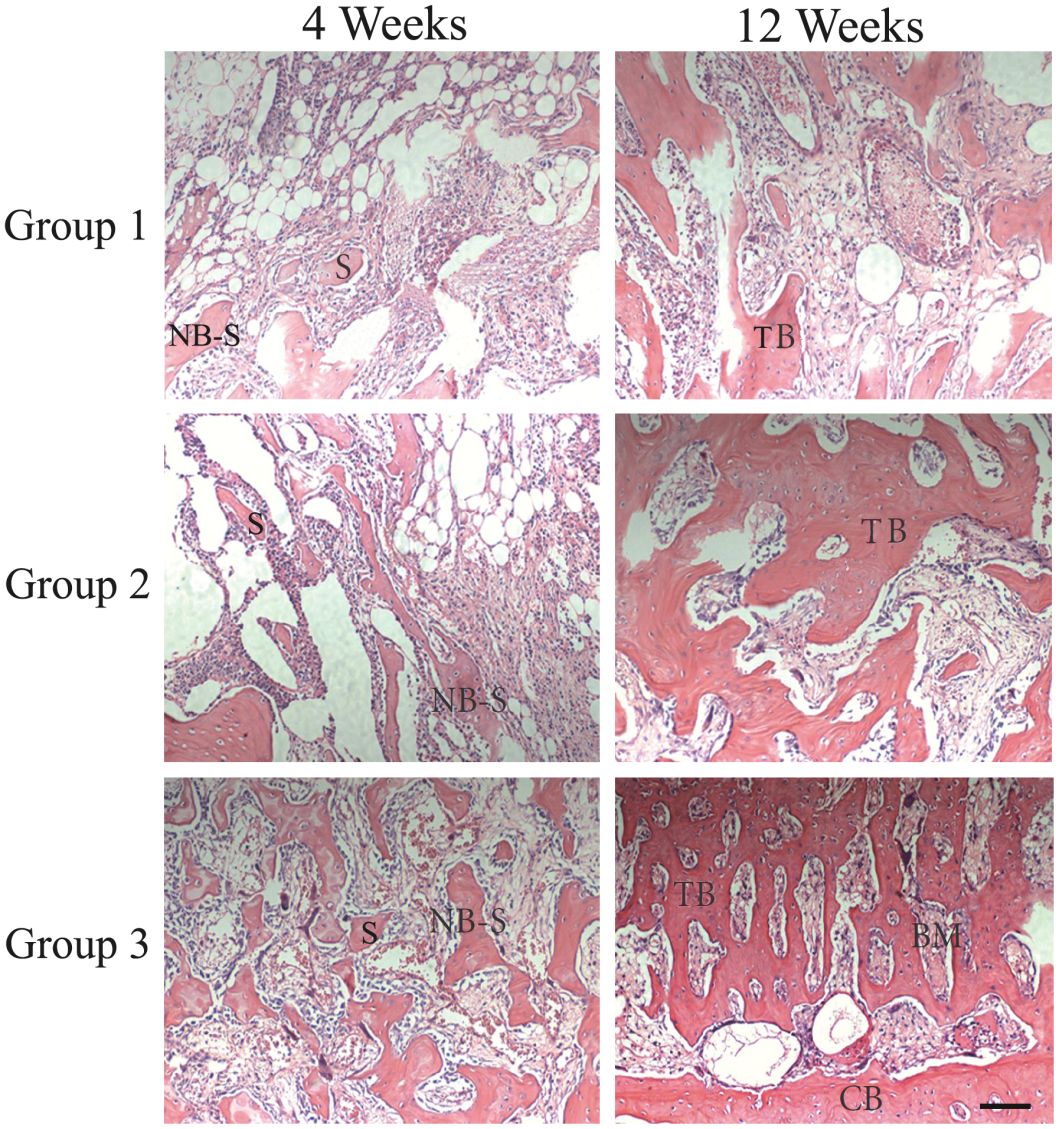
Histological observation in bone defect model. HE staining of the transverse bone defect sections in different groups at 4 weeks and 12 weeks post-surgery. Abbreviations used: scaffold (S); new bone from implanted scaffold (NB-S); bone marrow (BM); cortical bone (CB), trabecular bone (TB). Scale bar equals 400µm. (Group 1 referred to *β*-TCP group; Group 2 referred to ADSCs/*β*-TC group; Group 3 referred to Ad-CGRP-ADSCs/*β*-TCP group).

As shown in **Fig. 8**, at 4 weeks, new bone formation was observed in all implanted defects. In *β*-TCP and ADSCs/*β*-TCP groups, a small amount of the inflammatory cells can still be seen inside the scaffolds. But the scaffold had been replaced with neogenetic tissue, including a large number of new trabecular bones and fibrous tissue in Ad-CGRP-ADSCs/*β*-TCP group. At 12 weeks, the inflammatory reaction disappeared in all groups. In *β*-TCP and ADSCs/*β*-TCP groups, a few bone trabecular, part cortical bone and bone marrow could be seen inside the bone defects. However, in Ad-CGRP-ADSCs/*β*-TCP group, normal cortical bone, and bone marrow can be observed which indicated that the radical defect had recovered completely. Similar to that, the bone forming area calculated by Image Analysis System was 97.18±5.66% in Ad-CGRP-ADSCs/*β*-TCP group at 12 weeks which was significantly higher than *β*-TCP group (42.12±3.78%) and ADSCs/*β*-TCP group (60.58±5.29%) (*p*<0.05).

## Discussion

Genetically modified bone tissue engineering is an attractive approach with great potential for repairing bone defects that result from trauma, surgical resection, and congenital deformity corrections [3]–[5]. Many of these studies have focused on bone marrow mesenchymal stem cells. However, few related reports on adipose tissue derived stem cells (ADSCs) are available [20]. Adipose tissue has several advantages, including abundance and ease of acquisition, and is becoming a promising seed cell source [8]. Additionally, adenoviral vectors can transduce both dividing and non-dividing cells and provided prolonged target gene expression, high transfection efficiency, and low toxicity [21], [22]. In our study, ADSCs were chosen as donor cells, and adenoviral vectors were used for transduction. CGRP-transduced ADSCs can be transfected with high transduction efficiency, (approximately 81%), which demonstrates that transduction of ADSCs using an adenoviral vector is a feasible and efficient way to incorporate a foreign gene. Moreover, over-expression of CGRP was detected at a significantly higher level at 3 days and 7 days after transduction than the control group. Consequently, these results demonstrate that ADSCs and adenovirus-mediated gene targeting vectors are applicable to tissue engineering.

The calcitonin family of peptides has been extensively studied in bone over the past few years because of their effects on bone cells, especially osteoblasts. Some research has shown that CGRP increased the proliferation rate of osteoblasts [23], [24] and bone mass in transgenic mice over-expressing CGRP driven by an osteoblast-specific promoter [16]. Others observed that it could prevent bone loss when delivered systemically to ovariectomized rat [25] and CGRP-a knockout mice were osteopenic [17]. In this study, we also observed a morphological and characteristic change in ADSCs expressing CGRP. First, the morphology of CGRP-ADSCs appeared as heterogeneous population of spindle-shaped cells when the cells were no longer in colony growth mode. Second, long spindle-shaped cells dominated when growth was evenly distributed. However, untransduced ADSCs only formed a monolayer of flat, fibroblast-like cells. The CGRP-ADSCs were more proliferative and had a shorter doubling time compared to the untransduced ADSCs. These results demonstrated the CGRP genetically modified ADSCs have more vitality and more proliferative.

Although numerous investigators have identified the presence of CGRP receptors and observed CGRP stimulatory effects in osteoblasts, no previous studies have examined CGRP osteogenic effects of CGRP on MSCs. In our study, CGRP-ADSCs were induced to differentiate into osteogenic lineages using specific culture medium. Correspondingly, more ALP staining and increased ALP activity were detected in CGRP-ADSCs compared to the untransduced ADSCs (*p*<0.05) (Table 2). Furthermore, more and larger mineralized nodules formed in the transfected group compared to the control group. Finally, the CGRP- ADSCs treated with differentiation medium had a significant increase in the expression of the osteoblast-specific genes collagen I, BPG and OPN compared to control groups (Fig. 5), demonstrating successful osteogenic differentiation. Furthermore, implanted into bone defect model with scaffold in vivo, activity of bone regeneration in Ad-CGRP-ADSCs/*β*-TCP group displayed faster and more effective than *β*-TCP and ADSCs/*β*-TCP groups in various implantation periods (Fig. 7 and Fig. 8). Therefore, our study demonstrated that rat CGRP-ADSCs can be differentiated into pheonotypical and functional osteoblast cells. Currently, the mechanism of osteogenic differentiation has not been clarified. Possibly, CGRP-ADSCs might be stimulated by canonical Wnt signaling and inhibition of cell apoptosis [24], [26], or by modulation of the OPG/RANKL/RANK system [27], [28].

In summary, this study demonstrated that the adenovirus-mediated CGRP-ADSCs successfully differentiated into osteoblasts *in vitro* which maintained a high proliferative capacity and successfully secreted extracellular matrix, and also exhibited ideal seed cells for bone tissue engineering to repair bone defects *in vivo*. Hence, the novel CGRP transduced ADSCs represents effective and feasible seed cells which may provide a promising tissue engineering bone to treat bone loss problems in human cases.
